# Ultrasound study of carotid and cardiac remodeling and cardiac-arterial coupling in normal pregnancy and preeclampsia: a case control study

**DOI:** 10.1186/1471-2393-14-113

**Published:** 2014-03-25

**Authors:** Li-Jun Yuan, Yun-You Duan, Dan Xue, Tie-Sheng Cao, Ning Zhou

**Affiliations:** 1 Department of Ultrasound Diagnostics, Tangdu Hospital, Fourth Military Medical University, Xi’an 710038, China

**Keywords:** Preeclampsia, Ventriculo-arterial coupling, Ultrasound, Diastolic function, Arterial stiffness

## Abstract

**Background:**

Cardiovascular adaptions, such as cardiac and uterine spiral arterial remodeling, and aortic arterial stiffening during pregnancy have been extensively investigated, while the interactions between the elastic artery and the left ventricle are poorly understood. This study was to evaluate the cardiac-arterial coupling in both normal pregnancy and preeclampsia using ultrasound techniques.

**Methods:**

Twenty-three preeclamptic women with no antihypertensive treatment prior to admission, and 40 age- (27.2 ± 3.0 y vs. 29.1 ± 5.7 y, p = 0.0805) and gestational week- (35.6 ± 3.4 wk vs. 34.8 ± 3.6 wk, p = 0.3573) matched normotensive pregnant women were included. All women signed informed consent. All were nulliparas, had singleton pregnancies, and had no other risk factors for arterial stiffening. Carotid and cardiac ultrasound was performed using a MylabTwice ultrasound unit (Esaote, Italy). Cardiac and carotid remodeling and their associations were analyzed. Left ventriculo-carotid coupling was characterized by the ratio between the arterial elastance (Ea) and the left ventricular systolic elastance (Ees). Follow-up study was performed 16–20 months after parturition.

**Results:**

Left ventricular and carotid arterial remodeling was seen more frequently in preeclamptic women than in normal pregnant controls (96% vs. 40%, 82% vs. 48%, both p < 0.0001). The relative carotid arterial wall thickness showed no significant difference between the two groups. However, the carotid cross-sectional area, a surrogate for carotid arterial mass, was significantly greater in preeclampsia than that in normal controls (11.23 ± 0.17 mm^2^ vs. 8.58 ± 1.88 mm^2^, p < 0.00001). Carotid arterial stiffness and intima-media thickness correlated significantly with cardiac diastolic function parameters and blood pressures (p < 0.05). Both Ea and Ees were significantly greater in preeclampsia, compared with values in normal pregnant controls (Ea: 2.41 ± 0.57 mmHg/ml vs. 1.98 ± 0.46 mmHg/ml, p = 0.0005; Ees: 11.68 ± 9.51 m/s^2^ vs. 6.91 ± 6.13 m/s^2^, p = 0.002). However, there was no significant difference in the left ventriculo-carotid coupling index, Ea/Ees, between the two groups. Carotid remodeling persisted in both preeclamptic women and normal pregnant controls 16–20 months after parturition.

**Conclusions:**

Significant cardiac and carotid remodeling and similar left ventriculo-carotid coupling were observed in both preeclampsia and normal pregnancy. Carotid remodeling may persist postpartum. Further studies with larger populations are needed to confirm these findings.

## Background

Cardiovascular adaptions to pregnancy, such as cardiac and uterine spiral arterial remodeling, and aortic arterial stiffening, have been extensively investigated [1-3]. Our previous studies showed that carotid remodeling and arterial stiffening occurred in preeclampsia [4]. However, the associations between elastic arterial stiffness and cardiac morphology and function and cardiac-arterial coupling are poorly understood.

The recent development of high resolution ultrasound based on radio frequency signal allows us to assess regional arterial wall stiffness and intima-media thickness [5]. This study was designed to investigate the relationship between carotid arterial intima-media thickness and arterial stiffness, with cardiac morphology and function and left ventriculo-carotid coupling in both normal pregnancy and preeclampsia. Traditional echocardiography was performed and cardiac functional parameters were measured. Left common carotid artery ultrasound was performed with a MylabTwice ultrasound unit (Esaote, Italy) equipped with Automatic Quality Intima-Medial Thickness (QIMT) and (QAS) packages. Left ventricular and carotid remodeling, and left ventriculo-carotid coupling were analyzed.

## Methods

### Study population

Twenty-three consecutive severe preeclampsia Asian (Chinese) women with no antihypertensive treatment prior to admission, and 40 age- (29 ± 6 y vs. 27 ± 3 y, p = 0.081) and gestational week-matched (35.6 ± 3.4 wk vs. 34.8 ± 3.6 wk, p = 0.3573) consecutive normotensive pregnant women were included. There were no differences in maternal height and body surface area between the two groups (Table 1). All women were recruited from our routine prenatal clinic between January 2010 and July 2012. All were nulliparous, had singleton pregnancies, and had no other risk factors for arterial stiffening including smoking, sleep apnea, in vitro fertilization conception, diabetes, or hypercholesterolemia. Women with gestational hypertension and chronic hypertension were excluded. The study complied with the Declaration of Helsinki and the research protocol was approved by the Human Subjects Ethics Committee of our University. All women signed informed consent. The diagnosis of preeclampsia was based on the guidelines of the International Society for the Study of Hypertension in Pregnancy [6]. All measurements were performed by two investigators before noon in a quiet room with the subjects in a supine position. Five consecutive measurements were performed and then averaged as the final result for each patient.

**Table 1:** Demographic characteristics of normal pregnancy and preeclampsia

**Parameters**	**Normal pregnancy**	**Preeclampsia**	**P-Values**
	**(n = 40)**	**(n = 23)**	
**Maternal age, years**	27.2 ± 3.0	29.1 ± 5.7	0.0805
**Gestational age, weeks**	34.7 ± 3.6	35.6 ± 3.4	0.2929
**Maternal height, m**	1.60 ± 0.04	1.61 ± 0.04	0.1114
**Body surface area, m**^**2**^	1.64 ± 0.42	1.68 ± 0.10	0.6491
**Pregnancy BMI, kg/m**^**2**^	25.4 ± 2.4	27.8 ± 3.9	0.0054
**SBP, mmHg**	116.1 ± 8.6	151.4 ± 13.7	<0.00001
**DBP, mmHg**	75.4 ± 9.2	102.4 ± 8.3	<0.00001
**MAP, mmHg**	88.9 ± 7.9	118.8 ± 8.7	<0.00001
**Gestational week at delivery, week**	40 ± 1	37 ± 1	0.0263

### Blood pressure and hemodynamic measurements

Before ultrasound examination, brachial blood pressure (BP) measurements were taken using an oscillometric device (BP-203i, Colin Medical, Komaki, Japan) at 3-minute intervals for 20 minutes, and the average was taken as the casual BP level. Mean arterial pressure (MAP) was calculated as diastolic BP + [(systolic BP − diastolic BP)/3].

### Ultrasound examinations

#### Cardiac examination

All of the women underwent traditional echocardiography using a MylabTwice ultrasound unit (Esaote, Italy) equipped with a PA230 transducer. Cardiac output was calculated by the Teichholz formula using M-mode echocardiography [7]. Relative wall thickness (RWT) was defined as (interventricular diastolic septum thickness + posterior wall diastolic thickness)/left ventricular (LV) end-diastolic diameter. LV geometric patterns were determined by RWT and the left ventricular mass [8,9]. Total peripheral resistance (TPR, dyne.sec/cm^5^) was calculated as TPR = MAP (Kpa)/CO (L/min) × 80, where CO represents cardiac output.

#### Carotid examination

All of the women underwent left common carotid artery measurements with the MylabTwice ultrasound unit (Esaote, Italy) equipped with Automatic (QIMT) (Quality Intima-Media Thickness) and Quality Arterial Stiffness (QAS) (Quality Arterial Stiffness) packages. We used the vascular probe LA523 with a frequency of 12 MHz to measure carotid intima-media thickness (IMT), and the carotid diameter and carotid stiffness parameters: pulse wave velocity (PWV, m/s), augmentation index (AIx,%), and distensibility coefficient (DC, 1/KPa). α and β were measured and correlated with cardiac morphological and functional indices. To analyze the carotid remodeling, carotid cross-sectional area (mm^2^) was calculated according to the formula: [*π* × [(2 × IMT + D)/2]^2^ − *π* × (D/2)^2^] as a surrogate for carotid arterial mass, where D represents the internal diameter of the common carotid artery. Thirty non-pregnant age-matched women were selected to calculate the reference normal values for the carotid relative wall thickness and cross sectional area. The carotid geometry patterns of the pregnant women were classified into four types: normal, concentric remodeling, concentric hypertrophy, and eccentric hypertrophy.

#### Left ventriculo–arterial coupling analysis

The left ventriculo-arterial interaction was calculated from the ratio between carotid elastance (Ea) and end-systolic elastance (Ees). Ea is a measure of the arterial load and was calculated as: Ea = end-systolic pressure (mmHg)/stroke volume (SV, ml), where end-diastolic pressure could be replaced with systolic blood pressure. Ees was calculated noninvasively as LVOT peak flow velocity/LVOT acceleration time, where LVOT was the left ventricular outflow tract. Measurements of left ventriculo-carotid coupling were made in accordance with previously validated methodologies, and expressed as the ratio of Ea/Ees [10-12]. Left ventricular end-diastolic elastance (Ed, 1/ml) was calculated as: (E/e)/SV, where E was the transmitral early diastolic flow velocity, and e was the mitral annular velocity during diastole.

### Statistical analysis

All values were expressed as mean ± SD for continuous variables. Profiles in the two groups were compared using the unpaired Student’s t-test. Associations between the cardiac and arterial parameters were analyzed with Pearson’s correlation coefficient (r). A p-value < 0.05 was considered statistically significant. A power calculation performed before the study, using Ea/Ees, indicated that 21 participants were required for each group. According to the preliminary experiment, Ea/Ees results were (0.22 ± 0.1 mmHg•s^2^/ml•m) for the Preeclampsia group and (0.30 ± 0.1 mmHg•s^2^/ml•m) for the Normal group. The significance level was defined as alpha = 0.05. After calculation, the power was approximately 0.81. The difference we originally planned to detect was 0.08 mmHg•s^2^/ml•m. The statistical software package SPSS 12.0 (SPSS Inc., Chicago, IL) was used for all data analyses. Reproducibility and variability were confirmed in our previous study and good agreement and correlation were observed between measurements taken by the same observer and two different observers [4].

## Results

### Demographic characteristics of normal pregnancy and preeclampsia

The demographic characteristics of the subjects were shown in Table 1. The median birth weight was 2500 g (interquartile range: 2495 g, 2642 g) for preeclampsia and 3320 g (interquartile range: 3200 g, 3386 g) for normal pregnancy (p < 0.0001).

### Left ventricular morphological and functional remodeling

Diastolic function was significantly impaired in preeclamptic women compared with that in normal pregnant controls (Table 2). Left ventricular remodeling was seen more frequently in preeclamptic women (22/23, 96%) versus normal pregnant controls (16/40, 40%), p < 0.0001. Specifically, in the preeclampsia group, 14 (62%) showed concentric hypertrophy, 6 (26%) eccentric hypertrophy, and 2 (8%) concentric remodeling. In the control group, 20 (50%) showed concentric remodeling, and 4 (10%) showed concentric hypertrophy (Table 3).

**Table 2:** Diastolic and systolic function in normal pregnancy and preeclampsia

**Parameters**	**Normal pregnancy**	**Preeclampsia**	**P-values**
	**(n = 40)**	**(n = 23)**	
**Left heart size**			
LA size, mm	32.1 ± 3.1	35.1 ± 4.7	0.0034
LVEDD, mm	44.2 ± 3.1	46.0 ± 5.3	0.0913
**Diastolic function**			
E wave, cm/s	83.9 ± 15.1	93.0 ± 31.6	0.1361
A wave, cm/s	61.6 ± 14.2	75.7 ± 17.2	0.0011
E/A ratio	1.41 ± 0.31	1.24 ± 0.36	0.0658
Septal e wave, cm/s	13.8 ± 2.2	10.3 ± 3.4	<0.00001
Septal a wave, cm/s	12.0 ± 3.0	11.9 ± 3.2	0.8682
Septal e/a ratio	1.20 ± 0.28	0.90 ± 0.38	0.0007
Lateral e wave, cm/s	17.8 ± 2.9	14.3 ± 3.8	0.0001
Lateral a wave, cm/s	12.1 ± 3.3	12.6 ± 2.0	0.4946
Lateral e/a ratio	1.58 ± 0.49	1.15 ± 0.38	0.0010
Septal E/e ratio	6.06 ± 1.92	9.95 ± 4.39	<0.00001
Lateral E/e ratio	4.75 ± 1.56	6.77 ± 2.28	<0.00001
**Systolic function**			
Ejection fraction	0.66 ± 0.05	0.68 ± 0.10	0.2561
Fractional shortening (%)	36.6 ± 5.0	38.4 ± 8.2	0.2848

Notes: LA, left atrium; LVEDD, left ventricular end-diastolic dimension; E and A, transmitral flow velocity during early diastole and atrial contraction; e and a, mitral annular tissue velocities during early and late diastole.

**Table 3:** Hemodynamic changes and cardiac remodeling in normal pregnancy and preeclampsia

**Parameters**	**Normal pregnancy**	**Preeclampsia**	**P-values**
	**(n = 40)**	**(n = 23)**	
**Hemodynamic indices**			
HR, bpm	93 ± 15	89 ± 14	0.3100
CI, L/min/m^2^	2.99 ± 0.56	3.07 ± 0.72	0.5760
TPRI, (dynes × s^-1^ × cm^-5^)/m^2^	723.0 ± 123.3	894.1 ± 303.6	0.0023
**LV remodeling indices**			
RWT	0.44 ± 0.06	0.46 ± 0.09	0.3475
LVMI, g/m^2^	96.3 ± 17.8	110.9 ± 29.0	0.0005
**LV geometric pattern**			
Normal	16 (40)	1 (4)	<0.0001
Concentric remodeling	20 (50)	2 (8)	<0.0001
Eccentric hypertrophy	0 (0)	6 (26)	<0.0001
Concentric hypertrophy	4 (10)	14 (62)	<0.0001

Notes: Values are given as mean ± SD or no. of subjects (percentage). HR, heart rate; CI, cardiac index; TPRI, total peripheral resistance index; RWT, relative wall thickness; LVMI, left ventricular mass index; LV, left ventricle.

### Carotid morphological remodeling

The normal reference values for carotid RWT and cross sectional area for non-pregnant age-matched women were 0.12 ± 0.03 and 8.70 ± 2.07 mm^2^, respectively. Carotid RWT showed no significant difference between preeclamptic women and normal pregnant controls (0.11 ± 0.03 vs. 0.10 ± 0.02, p = 0.0835). However, the carotid cross-sectional area, a surrogate for carotid arterial mass, was significantly greater in preeclamptic women compared with that in normal pregnant controls (11.23 ± 0.17 mm^2^ vs. 8.58 ± 1.88 mm^2^, p < 0.00001). Carotid arterial remodeling was seen more frequently in preeclamptic women than in normal pregnant controls (82% vs. 48%, p < 0.0001). Specifically, 35% of preeclamptic women showed concentric hypertrophy and 47% showed eccentric hypertrophy. Eccentric hypertrophic morphology was seen more frequently in the normal pregnant control group compared with preeclamptic women (79% vs. 57%, p < 0.01). Carotid intima-media thickness as well as stiffness parameters correlated well with cardiac diastolic function parameters and blood pressures (Table 4).

**Table 4:** Correlations of carotid IMT and stiffness parameters with blood pressures and cardiac function

**Parameters**	**PWV**	**DC**	**AIx**	**α**	**β**	**IMT**	**TPR**
	**(m/s)**		**(%)**			**(μm)**	**(dynes/second/cm **^**-5**^**)**
**LA (mm)**	r = 0.1220	r = −0.0655	r = 0.2211	r = 0.0680	r = 0.0714	r = 0.0342	r = −0.1696
	ns	ns	ns	ns	ns	ns	ns
**RWT**	r = 0.4569	r = −0.2445	r = −0.0804	r = 0.4652	r = 0.4652	r = 0.0451	r = 0.0978
	p = 0.0002	ns	ns	p = 0.0002	p = 0.0002	ns	ns
**E/Septal e**	r = 0.0015	r = −0.0349	r = 0.4211	r = −0.0982	r = −0.0984	r = 0.2279	r = 0.1162
	ns	ns	p = 0.0026	ns	ns	ns	ns
**E/Lateral e**	r = 0.0555	r = −0.1200	r = 0.4439	r = −0.0730	r = −0.0733	r = 0.1625	r = 0.1814
	ns	ns	p = 0.0012	ns	ns	ns	ns
**Septal e (cm/s)**	r = −0.1814	r = 0.0906	r = −0.4401	r = −0.0510	r = −0.0472	r = −0.2588	r = −0.3995
	ns	ns	p = 0.0014	ns	ns	p = 0.0478	p = 0.0017
**Lateral e (cm/s)**	r = −0.3768	r = 0.3135	r = 0.3440	r = −0.2140	r = −0.2099	r = −0.1262	r = −0.4338
	p = 0.0028	p = 0.0031	p = 0.0135	ns	ns	ns	p = 0.0005
**Septal e/a**	r = −0.3908	r = 0.2327	r = −0.2229	r = −0.2857	r = −0.2808	r = −0.2371	r = −0.2292
	p = 0.0022	ns	ns	p = 0.0283	p = 0.02312	ns	ns
**Lateral e/a**	r = −0.4665	r = 0.3135	r = −0.1709	r = −0.2140	r = −0.3946	r = −0.0944	r = −0.1471
	p = 0.0002	p = 0.0139	ns	ns	p = 0.0018	ns	ns
**LVMI (g/m**^**2**^**)**	r = 0.0005	r = −0.0553	r = 0.2383	r = −0.0613	r = −0.0609	r = 0.0895	r = 0.0645
	ns	ns	ns	ns	ns	ns	ns
**CI (L/min/m**^**2**^**)**	r = 0.0355	r = −0.8839	r = −0.2129	r = 0.0357	r = 0.0343	r = 0.0186	r = −0.5894
	ns	ns	ns	ns	ns	ns	p < 0.0001
**SBP (mmHg)**	r = 0.5552	r = −0.4973	r = 0.5749	r = 0.2855	r = 0.2847	r = 0.3680	r = 0.4462
	p < 0.0001	p < 0.0001	p < 0.0001	p = 0.0257	p = 0.0261	p = 0.038	p = 0.0004
**DBP (mmHg)**	r = 0.3731	r = −0.2939	r = 0.6696	r = 0.0545	r = 0.0482	r = 0.3833	r = 0.4865
	p = 0.0031	p = 0.0215	p < 0.0001	ns	ns	p = 0.0025	p < 0.0001
**PP (mmHg)**	r = 0.5817	r = −0.5759	r = 0.1817	r = 0.4863	r = 0.4932	r = 0.1953	r = 0.2116
	p < 0.0001	p < 0.0001	ns	p < 0.0001	p < 0.0001	ns	ns
**MAP (mmHg)**	r = 0.4631	r = −0.3906	r = 0.6523	r = 0.1558	r = 0.1518	r = 0.3891	r = 0.4842
	p = 0.0002	p = 0.0019	p < 0.0001	ns	ns	p = 0.0021	p < 0.0001

Notes: PWV, pulse-wave velocity; DC, distensibility coefficient; AIx, augmentation index; IMT, intima-media thickness; TPR, total peripheral resistance; LA, left atrium; E, transmitral flow velocity during early diastole; e and a, mitral annular tissue velocities during early and late diastole; LVMI, left ventricular mass index; CI, cardiac index; SBP, DBP and MAP, systolic, diastolic and mean arterial blood pressure.

### Cardiac-arterial coupling

Ea, EaI (Ea adjusted for body area), Ees and Ed were all significantly greater in preeclampsia compared with normal pregnant controls (Ea: 2.41 ± 0.57 mmHg/ml vs. 1.98 ± 0.46 mmHg/ml, p = 0.0005; EaI: 1.32 ± 0.36 mmHg/ml · m^2^ vs. 1.10 ± 0.36 mmHg/ml · m^2^, p = 0.0025; Ees: 11.68 ± 9.51 m/s^2^ vs. 6.91 ± 6.13 m/s^2^, p = 0.002; Ed: 0.15 ± 0.07 ml^-1^ vs. 0.11 ± 0.03 ml^-1^, p = 0.0008). Ea and EaI were significantly correlated with left ventricular relative wall thickness, diastolic functional indices, blood pressures, and total peripheral resistance. Ees was closely correlated with left ventricular diastolic function parameters and blood pressures. Ed was significantly correlated with diastolic functional indices and total peripheral resistance. Ea/Ees was significantly correlated with the cardiac output index (Table 5). Ea/Ees showed no significant difference between the two groups (0.27 ± 0.11 mmHg•s^2^/ml•m vs. 0.29 ± 0.16 mmHg•s^2^/ml•m, p = 0.6986).

**Table 5:** Correlations of arterial ventricular coupling indices with blood pressures and cardiac function

**Parameters**	**Ea**	**EaI**	**Ees**	**Ea/Es**	**Ed**
	**mmHg/ml**	**mmHg/ml/m**^**2**^	**m/s**^**2**^	**mmHg · s**^**2**^**/ml · m**	**(1/ml)**
**LA (mm)**	r = −0.1349	r = −0.0655	r = 0.1488	r = −0.8881	r = 0.1052
	ns	ns	ns	ns	ns
**RWT**	r = 0.3103	r = 0.2974	r = 0.1980	r = 0.0059	r = −0.1507
	p = 0.0149	p = 0.0210	ns	ns	ns
**E/Septal e**	r = 0.0000	r = −0.0371	r = 0.1656	r = −0.1309	r = 0.8860
	ns	ns	ns	ns	p < 0.0001
**E/Lateral e**	r = 0.0253	r = −0.0090	r = 0.1064	r = −0.0425	r = 0.6280
	ns	ns	ns	ns	p < 0.0001
**Septal e (cm/s)**	r = −0.2166	r = −0.3183	r = −0.3371	r = 0.1372	r = −0.6746
	ns	p = 0.0149	p = 0.0090	ns	p < 0.0001
**Lateral e (cm/s)**	r = −0.3768	r = −0.3798	r = −0.3380	r = 0.0037	r = −0.2941
	p = 0.0030	p = 0.0030	p = 0.0083	ns	p = 0.0250
**Septal e/a**	r = −0.3619	r = −0.4373	r = −0.3410	r = −0.0668	r = −0.0473
	p = 0.0052	p = 0.0007	p = 0.0088	ns	ns
**Lateral e/a**	r = −0.3225	r = −0.2923	r = −0.1906	r = −0.1138	r = −0.2712
	p = 0.0127	p = 0.0260	ns	ns	p = 0.0413
**LVMI (g/m**^**2**^**)**	r = −0.3179	r = −0.3033	r = 0.0202	r = −0.2037	r = 0.0838
	p = 0.0141	p = 0.0195	ns	ns	ns
**CI (L/min/m**^**2**^**)**	r = −0.3146	r = −0.2487	r = 0.1737	r = −0.3674	r = −0.1230
	p = 0.0152	ns	ns	p = 0.0042	ns
**SBP (mmHg)**	r = 0.5457	r = 0.5542	r = 0.4520	r = −0.1216	r = 0.2386
	p < 0.0001	p < 0.0001	p = 0.0003	ns	ns
**DBP (mmHg)**	r = 0.4427	r = 0.4490	r = 0.2983	r = −0.0602	r = 0.2525
	p = 0.0004	p = 0.0004	p = 0.0206	ns	ns
**PP (mmHg)**	r = 0.4695	r = 0.5080	r = 0.4830	r = −0.1575	r = 0.1237
	p = 0.0002	p < 0.0001	p < 0.0001	ns	ns
**MAP (mmHg)**	r = 0.5002	r = −0.5080	r = 0.3734	r = −0.0885	r = 0.2540
	p < 0.0001	p < 0.0001	p = 0.0033	ns	ns
**TPR (dynes/second / cm **^**-5**^**)**	r = 0.4650	r = 0.5579	r = 0.1490	r = 0.1340	r = 0.3218
	p = 0.0002	p < 0.0001	ns	ns	0.0146

Notes: Ea, arterial elastance; EaI: Ea adjusted for body mass index; Ees, end-systolic elastance; Ed, left ventricular end-diastolic elastance; LA, left atrium; RWT, relative wall thickness; E, transmitral Doppler flow velocity during early diastole; Septal e and lateral e, mitral annular velocity during early diastole on septal and lateral sides, respectively, by tissue Doppler; Septal a and lateral a, mitral annular velocity during atrial contraction on septal and lateral sides, respectively, by tissue Doppler; LVMI, left ventricular mass index; CI, cardiac index; SBP, systolic blood pressure; DBP, diastolic blood pressure; PP, pulsed pressure; MAP, mean arterial pressure; TPR, total peripheral resistance.

### Follow-up study

Seven preeclampsia women and 7 normal pregnant women were followed up for 16–20 months after parturition (Tables 6, 7). Using the normal non-pregnant age-matched women’s reference values, which were 0.12 for relative carotid arterial wall thickness and 8.70 mm^2^ for carotid luminal cross sectional area, the cardiac morphological and functional parameters showed no significant difference between preeclamptic women and normal pregnant controls (Table 6). The majority of the cardiac remodeling resolved, but the carotid remodeling persisted in both preeclamptic women and normal pregnant controls, predominantly in those with eccentric hypertrophy (4/7 and 7/7).

**Table 6:** Comparison of diastolic and systolic function in normal pregnancy and preeclampsia postpartum

**Parameters**	**Normal pregnancy postpartum**	**Preeclampsia postpartum**	**P-values**
	**(n = 7)**	**(n = 7)**	
**Brachial arterial pressures**			
SP, mmHg	107 ± 14	127 ± 17	0.0327
DP, mmHg	77 ± 8	82 ± 9	0.2371
MAP, mmHg	87 ± 9	97 ± 12	0.0880
**Left heart size**			
LA size, mm	28.4 ± 1.9	27.6 ± 2.9	0.5293
LVEDD, mm	42.9 ± 1.8	44.0 ± 3.8	0.4874
**Diastolic function**			
E/A ratio	1.65 ± 0.30	1.42 ± 0.36	0.2128
Septal e/a ratio	1.51 ± 0.22	1.32 ± 0.31	0.2204
Lateral e/a ratio	1.42 ± 0.45	1.45 ± 0.30	0.1554
**Systolic function**			
Ejection fraction	0.70 ± 0.03	0.69 ± 0.03	0.6278
Fractional Shortening (%)	39.3 ± 1.9	38.7 ± 2.1	0.5984
CI, L/min/m^2^	3.0 ± 0.5	2.7 ± 0.8	0.4355
**Hemodynamics**			
TPRI, (dynes × s^-1^ × cm^-5^)/m^2^	904.9 ± 203.7	1064.2 ± 396.7	0.3088

**Table 7:** Comparison of cardiac and carotid remodeling between normal pregnancy and preeclampsia postpartum

**Parameters**	**Normal pregnancy postpartum**	**Preeclampsia postpartum**	**P values**
	**(n = 7)**	**(n = 7)**	
LV RWT	0.36 ± 0.02	0.39 ± 0.06	0.2519
LVMI, g/m^2^	74.2 ± 15.3	81.7 ± 14.0	0.3790
Carotid RWT	0.11 ± 0.03	0.12 ± 0.05	0.4771

Notes: LV RWT, left ventricular relative wall thickness; LVMI, left ventricular mass index.

## Discussion

Our study showed that remodeling occurred both in the carotid artery and in the heart in normal pregnant and preeclamptic women. Maternal carotid intima-medial thickness and arterial stiffness parameters were closely correlated with the cardiac morphological and diastolic functional indices. However, the arterial ventricular coupling index showed no significant difference between the two groups, possibly due to the complex hemodynamic states in patients with preeclampsia. Further studies with subgroups classified according to hemodynamic patterns are needed to confirm the findings. To our knowledge, our study is the first to comprehensively explore both cardiac and carotid remodeling and left ventriculo-carotid coupling in preeclamptic women.

### Cardiac and carotid remodeling in normal pregnancy and preeclampsia

In our study patients, cardiac concentric hypertrophy was frequently observed in the preeclamptic women, similar to the results of a study by Melchiorre et al. [13]. In their study, the preeclamptic women were divided into two groups: one group with normal diastolic function and another group with diastolic dysfunction. The results showed that concentric and eccentric remodeling were seen in preeclamptic women with normal left ventricular diastolic function, while concentric hypertrophy was seen in preeclamptic women with left ventricular diastolic dysfunction. The cardiac concentric hypertrophy in these preeclamptic women might have resulted from the increased left ventricular wall thickness due to elevated total peripheral resistance, elevated blood pressure, and an absence of increased left ventricular end-diastolic dimensions.

In contrast to the cardiac remodeling, eccentric carotid arterial hypertrophy was seen more frequently in preeclamptic women (74%) than in normal pregnant controls (43%), possibly as a result of the greater increased carotid internal diameter compared with the incremental carotid intima-media thickness. The difference between the cardiac and carotid remodeling pattern distribution in preeclamptic women might be explained by the difference in afterload. For the left ventricle, it has been shown that aortic arterial stiffness is significantly increased in preeclampsia [14]; while for the carotid artery, distal cerebral arterial resistance has been shown to significantly decrease as compensation in preeclampsia [15]. These differences in afterload might account for the variety of cardiac and carotid remodeling patterns in these subjects.

### Cardiac-arterial coupling in normal pregnancy and preeclampsia

PWV has a strong correlation with cardiovascular events and all-cause mortality [16-19], and was recognized by the European Society of Hypertension as integral to the diagnosis and treatment of hypertension [20]. In our study, carotid PWV correlated closely with left ventricular relative wall thickness, septal and LV lateral e/a ratio, and blood pressure, indicating that the higher the blood pressure in pregnancy, the worse the diastolic function and the stiffer the carotid artery. DC is the fractional change in cross-sectional area relative to the change in arterial pressure. Interestingly, DC showed a close correlation with the lateral e and lateral e/a ratio of the mitral annulus, but not the septal e and septal e/a ratio. LV lateral e and lateral e/a ratio are less influenced by the right ventricle compared with septal e and septal e/a ratio, although it has been demonstrated that the septal mitral annulus provides better diagnostic utility [21]. AIx is a parameter based on the analysis of the pressure waveform and represents the ratio of augmented pressure (attributed to wave reflection) to pulse pressure. In our study, AIx was significantly associated with systolic, diastolic, and mean arterial pressures, but not with the pulse pressure. Our results also showed that AIx was the only carotid arterial stiffness index that correlated with the total peripheral resistance. α and β were calculated from changes in vessel diameter and blood pressure, and were closely correlated with the left ventricular relative wall thickness, systolic and pulse pressure, but not with the diastolic and mean arterial pressures. As expected, total peripheral resistance was closely related to the cardiac output index and blood pressures.

It has been shown that Ea/Ees could better reflect cardiac-arterial coupling. Ea itself reflects the artery’s ability to expand and contract in synchrony with cardiac pulsation and relaxation. We found that Ea was significantly increased in our patients with preeclampsia, implying that the ability of the artery to respond to the cardiac activities decreased in these subjects. Ees represents end-systolic elastance, which provides an index of myocardial contractility. This index is relatively insensitive to changes in preload, afterload, and heart rate and is an improved index of systolic function over other hemodynamic parameters like ejection fraction, cardiac output, and stroke volume. Our patients with preeclampsia showed both increased Ees and impaired left ventricular diastolic function, which may increase myocardial oxygen consumption, limiting myocardial perfusion reserve [22]. By characterizing both the ventricular and arterial systems in terms of pressure and stroke volume, it is possible to study arterial-ventricular coupling, i.e., the interaction between the heart and the arterial system. Interestingly, our results showed no significant difference in Ea/Ees between the preeclamptic and normal pregnant women, which might be related to the complex hemodynamic characteristics in patients with preeclampsia.

### Cardiac and carotid changes after parturition

Our follow-up results showed that the cardiac morphology and functional parameters in preeclamptic women returned to the level of the normal controls 16–20 months after parturition. However, carotid remodeling persisted in both preeclamptic women and normal pregnant women, suggesting peripheral vascular remodeling during pregnancy persists longer than cardiac remodeling. The implications of these findings need further exploration.

### Limitations

The first limitation of this study was that we included limited cardiac diastolic and systolic function indices. For example, we did not measure pulmonary venous flow indices and longitudinal systolic function indices because the four chamber views were difficult to display satisfactorily in late gestational women. The second limitation is the small number of patients, which was because we assessed only late gestational preeclamptic women with no antihypertensive treatment prior to admission. We assume that the significant difference between the preeclamptic women and the normal pregnant controls with respect to carotid arterial stiffness, cardiac diastolic function, and arterial-ventricular correlations would exist even in a larger study population. The final limitation is that the carotid measurements were performed in a supine position, which might be associated with significant vascular changes. It would be interesting to observe the vascular hemodynamic changes with different positions in these pregnant women in future studies.

## Conclusions

In conclusion, this study implies a close correlation between carotid arterial stiffness and early left ventricular dysfunction. Arterio-ventricular coupling showed no significant difference between preeclamptic and normal pregnancy, which might be due to the complex hemodynamic patterns in preeclampsia. Further studies with larger populations and subgroups classified according to hemodynamic patterns are needed to confirm these findings. Carotid remodeling persists 16–20 months after parturition in both preeclamptic and normal pregnant women. The implications of this finding need to be further explored by observing different carotid arterial remodeling types comparing preeclamptic and normal pregnancy in larger groups.

## Abbreviations

QIMT: Quality Intima-Media Thickness; QAS: Quality arterial stiffness; BP: Blood pressure; MAP: Mean arterial pressure; LV: Left ventricle; RWT: Relative wall thickness; TPR: Total peripheral resistance; PWV: Pulse wave velocity; AIx: Augmentation index; DC: Distensibility coefficient; SV: Stroke volume; LVOT: Left ventricular outflow tract; LVMI: Left ventricular mass index; LA: Left atrium; LVEDD: Left ventricular end-diastolic dimension.

## Competing interests

The authors declare that they have no competing interests.

## Authors’ contributions

The contributions of individual authors to this paper were as follows: YLJ, XD, and DYY participated in: 1. the conception and design, acquisition, analysis and interpretation of data, development of the hypothesis and research plan, establishment of methodology; 2. drafting of the manuscript and critical revision of the manuscript for intellectual content, and 3. final approval of the version to be published. CTS and ZN were involved in: 1. acquisition of data, analysis and interpretation of data; 2. assistance with revising the manuscript, and 3. final approval of the version to be published. All authors read and approved the final manuscript.

## Pre-publication history

The pre-publication history for this paper can be accessed here:

http://www.biomedcentral.com/1471-2393/14/113/prepub
